# Skin Metastasis of an Occult Primary Bronchopulmonary Disease: A Case Report and Literature Review

**DOI:** 10.7759/cureus.115948

**Published:** 2026-09-08

**Authors:** Hadil Rafi, Soufiane Berhili, Ahmed BenSghier, Mohamed Moukhlissi, Loubna Mezouar

**Affiliations:** 1 Radiotherapy, Centre Hospitalier Universitaire Mohammed VI, Oujda, MAR; 2 Radiation Oncology, Faculty of Medicine and Pharmacy, Mohammed First University, Oujda, MAR; 3 Radiation Oncology, Centre Hospitalier Universitaire Mohammed VI, Oujda, MAR; 4 Radiotherapy, Mohammed VI University Hospital, Oujda, MAR

**Keywords:** cutaneous metastasis, immunohistochemistry, lung adenocarcinoma, napsin a, occult primary tumor, palliative chemotherapy, ttf-1, whole-brain radiotherapy

## Abstract

Cutaneous metastases of pulmonary origin are rare but clinically significant, typically indicating advanced disease with a poor prognosis. They may occasionally reveal an occult lung cancer, as illustrated by the case reported here.

We report the case of a 78-year-old man, a former heavy smoker, who presented with a rapidly growing, ulcerated, exophytic nodule on the anterior chest wall. Skin biopsy revealed a poorly differentiated adenocarcinoma, with negative thyroid transcription factor-1 (TTF-1) and Napsin A staining, raising diagnostic difficulty regarding the primary origin. Chest CT subsequently identified a 4.1 cm tumor of the right lower lobe, and bronchoscopic biopsy confirmed a bronchial adenocarcinoma. Staging revealed disseminated disease (cT2b cN1 cM1c, Stage IVB) with cerebral, adrenal, and bone metastases. A multidisciplinary tumor board recommended whole-brain radiotherapy (30 Gy in 10 fractions) combined with palliative chemotherapy (cisplatin-gemcitabine), with stable disease observed at six months of follow-up.

This case underlines the limitations of TTF-1 and Napsin A negativity in excluding a pulmonary origin, particularly in poorly differentiated tumors, and highlights the value of an integrated clinical, radiological, morphological, and immunohistochemical approach. Management of extensively disseminated disease remains essentially palliative; surgery has no impact on survival and is reserved for palliative or diagnostic indications.

## Introduction

Lung cancer is the leading cause of cancer-related death worldwide, accounting for approximately 1.8 million deaths annually [1]. This poor prognosis is largely explained by late diagnosis, as most patients already present with metastatic disease at the time the primary tumor is discovered. Adenocarcinoma constitutes the predominant histological subtype of non-small cell lung carcinomas, accounting for 40% to 50% of cases [1], and metastatic spread most commonly involves lymph nodes, the pleura, the contralateral lung, brain, bone, and adrenal glands.

Cutaneous involvement, by contrast, remains rare and often underrecognized. Cutaneous metastases occur in approximately 3% to 10% of all neoplasms and in 1% to 12% of lung cancers [1]. They are most often discovered after the diagnosis of the primary tumor; however, their appearance can be synchronous, and in some cases, metastasis constitutes the first clinical manifestation, described as "early," a scenario that poses a major diagnostic challenge, as no primary tumor is yet known.

Clinically, these lesions typically consist of one to several hard, immobile subcutaneous nodules ranging in size from 0.5 to 10 cm, most frequently of adenocarcinoma histology, followed by squamous cell carcinoma [2]. These cutaneous metastases most often result from hematogenous dissemination rather than direct contiguous extension of the primary tumor, which explains their possible occurrence at sites distant from the thoracic cavity [2]. The diagnosis is based primarily on histological analysis supplemented by immunohistochemistry; however, as illustrated in this case, classic pulmonary markers can be misleading, complicating identification of the primary origin. Management is most often palliative in nature, and the prognosis remains poor, with generally short survival [3].

In this article, we present the case of a metastatic pulmonary adenocarcinoma discovered fortuitously during the evaluation of a cutaneous metastasis, along with a review of the literature.

## Case presentation

A 78-year-old man with a history of chronic smoking (thirty pack-years, quit one year before presentation) and epilepsy under treatment consulted a dermatologist for a rapidly growing, painless lesion on his anterior chest. Clinical examination revealed a rounded, exophytic, tumorous cutaneous nodule on the anterior chest. It measured approximately 3.5 by 2.5 cm and featured a shiny surface, a central necrotic ulceration, hemorrhagic crusts, and a peripheral erythematous halo (Figure 1). The lesion was complicated by local pain and superficial infection, both managed with local wound care, along with minimal bleeding without hemodynamic repercussion.

**Figure 1:**
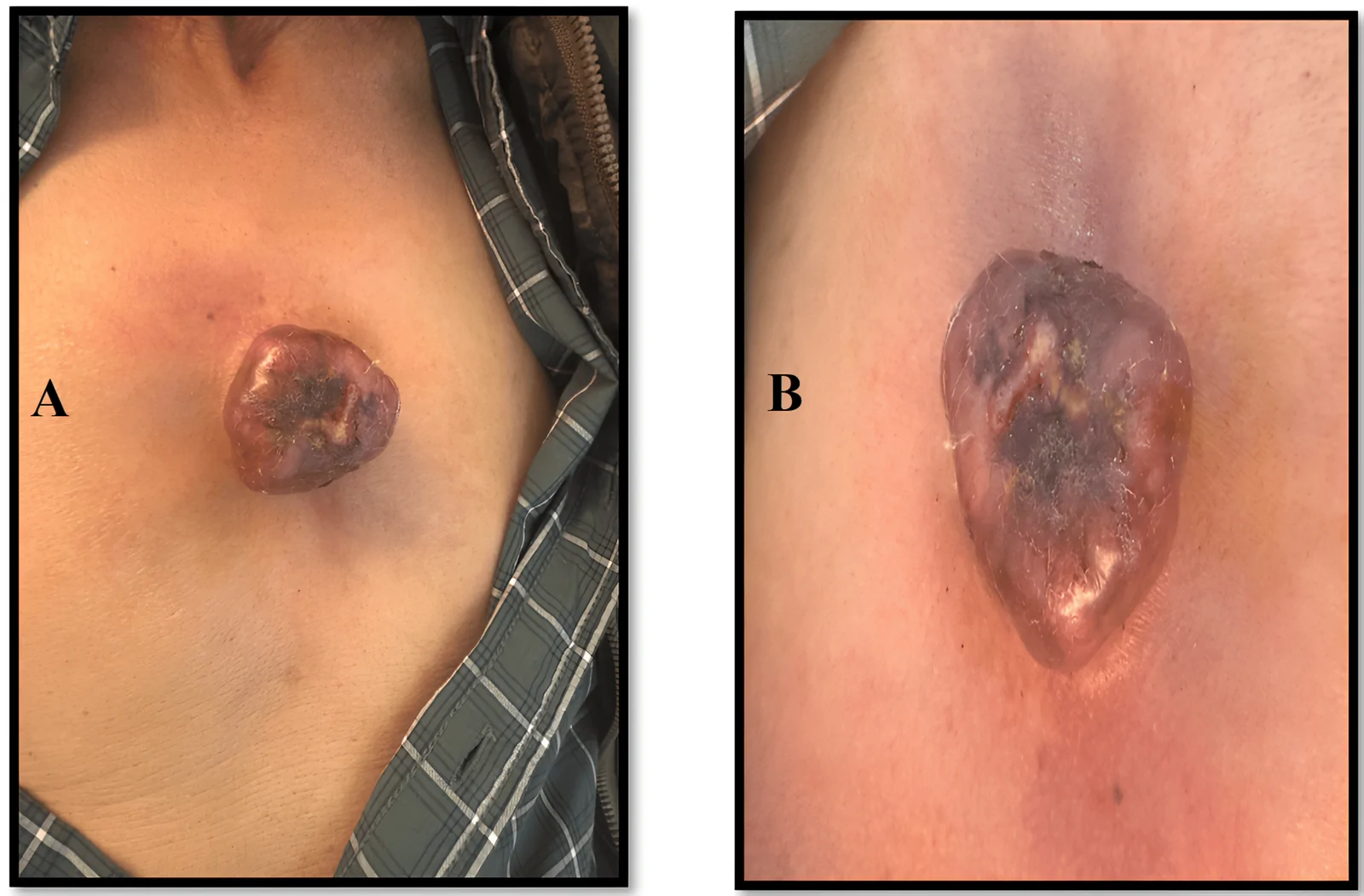
Clinical presentation of cutaneous metastasis of bronchopulmonary origin (A) Gross appearance of the cutaneous metastatic lesion on the anterior chest wall (B) Close-up view showing the exophytic and ulcerated appearance of the lesion

The patient was afebrile but had a general malaise, with no signs of spontaneous improvement. The rest of the clinical examination was unremarkable, and no respiratory abnormalities were found.

A biopsy of the nodule was performed, revealing a nodular cutaneous lesion covered by moderately acanthotic epidermis surmounted by compact orthokeratotic hyperkeratosis with an inflammatory border. Histological examination was consistent with a secondary localization of a poorly differentiated adenocarcinoma of pulmonary origin, despite negative results for thyroid transcription factor-1 (TTF-1) and Napsin A markers (Figure 2).

**Figure 2:**
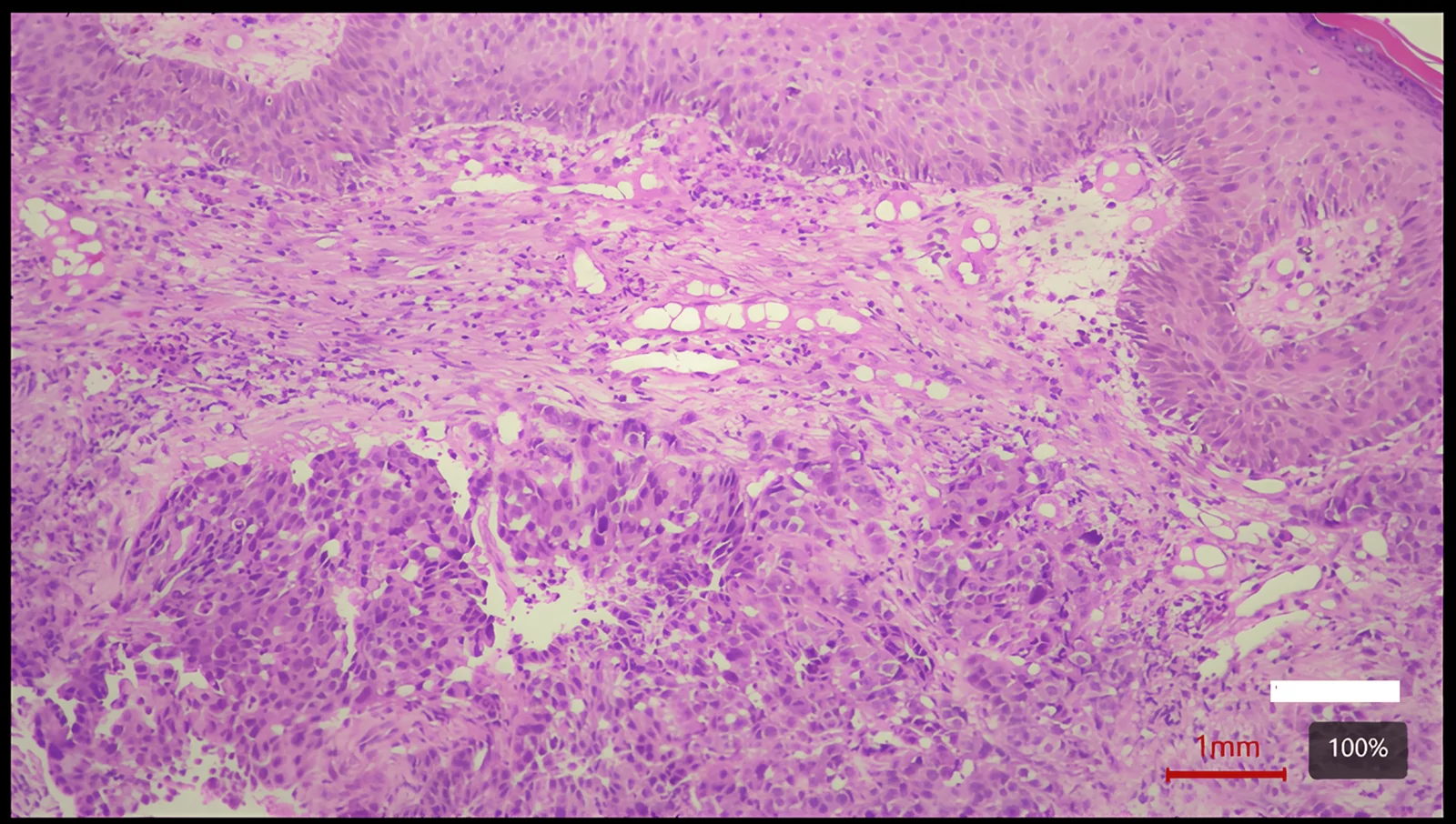
Anatomopathological section showing a dermis invaded by a poorly differentiated adenocarcinomatous proliferation made up of trabeculae, cords, and masses sometimes hollowed out by lumens.

The thoraco-abdomino-pelvic CT scan showed a parenchymal tumor process in the right lower lobe with a metastatic appearance, measuring 4.1 cm in its largest axis (Figure 3).

**Figure 3:**
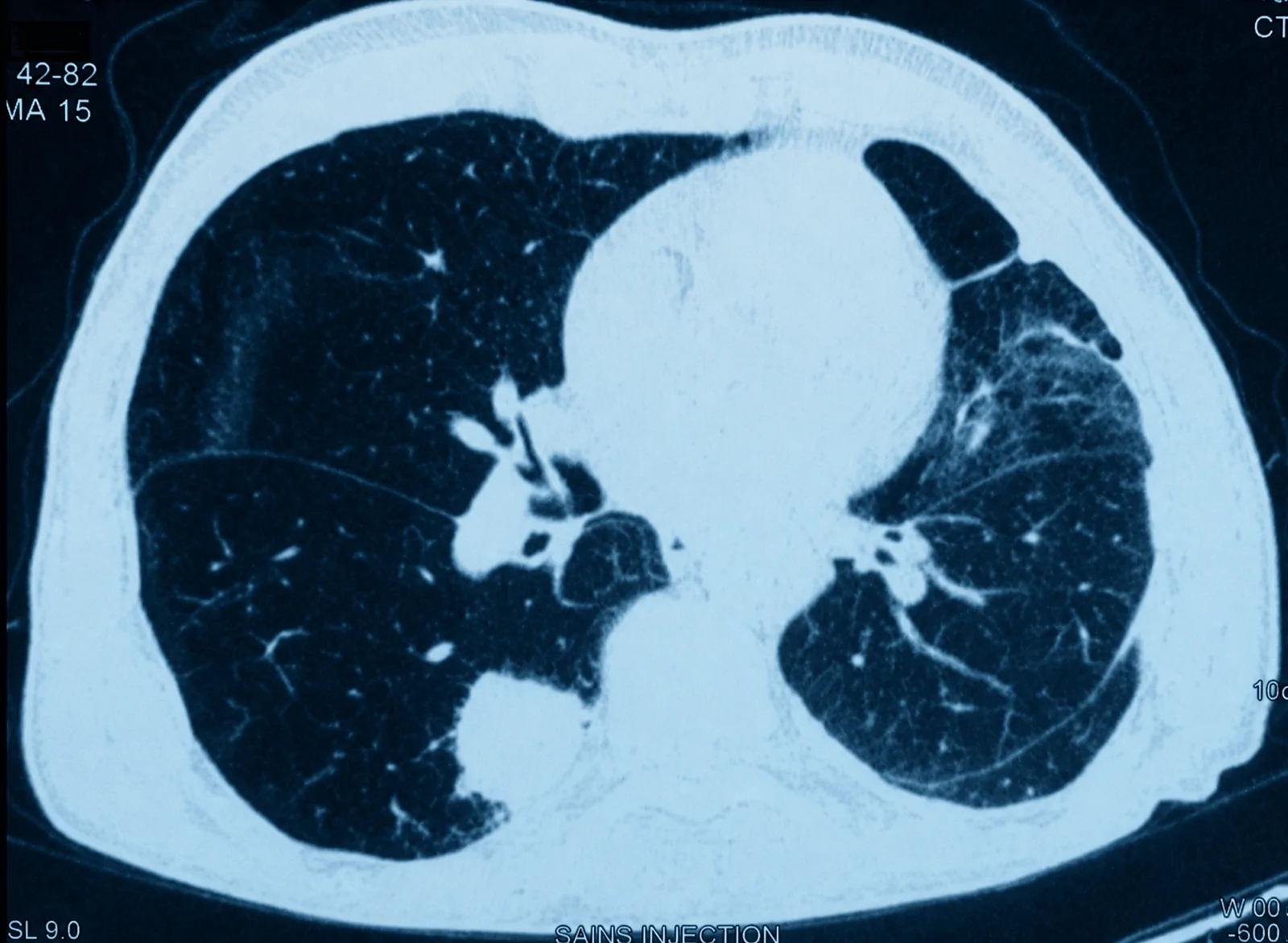
An axial thoracic section of the right lung showing a parenchymal tumor process in the lower lobe of the right lung.

Bronchoscopy revealed a thickening at the level of the intersegmental spur of the basal pyramid; the anatomopathological study of this lesion also favored a bronchial adenocarcinoma.

The staging workup (cerebral-cervico-thoraco-abdomino-pelvic CT scan) revealed other secondary locations, including cerebral, adrenal, and bone metastases, but all were clinically asymptomatic. The patient had an Eastern Cooperative Oncology Group (ECOG) performance status of 2. Staging corresponded to cT2b cN1 cM1c (Stage IVB) according to the 8th TNM classification. No molecular testing or programmed death-ligand 1 (PD-L1) assessment was performed.

Given this picture of metastatic disease, the therapeutic decision adopted according to the multidisciplinary tumor board (MTB) was a combined approach. This included whole-brain radiotherapy (WBRT) of 30 Gy in 10 fractions of 3 Gy per fraction (Figure 4).

**Figure 4:**
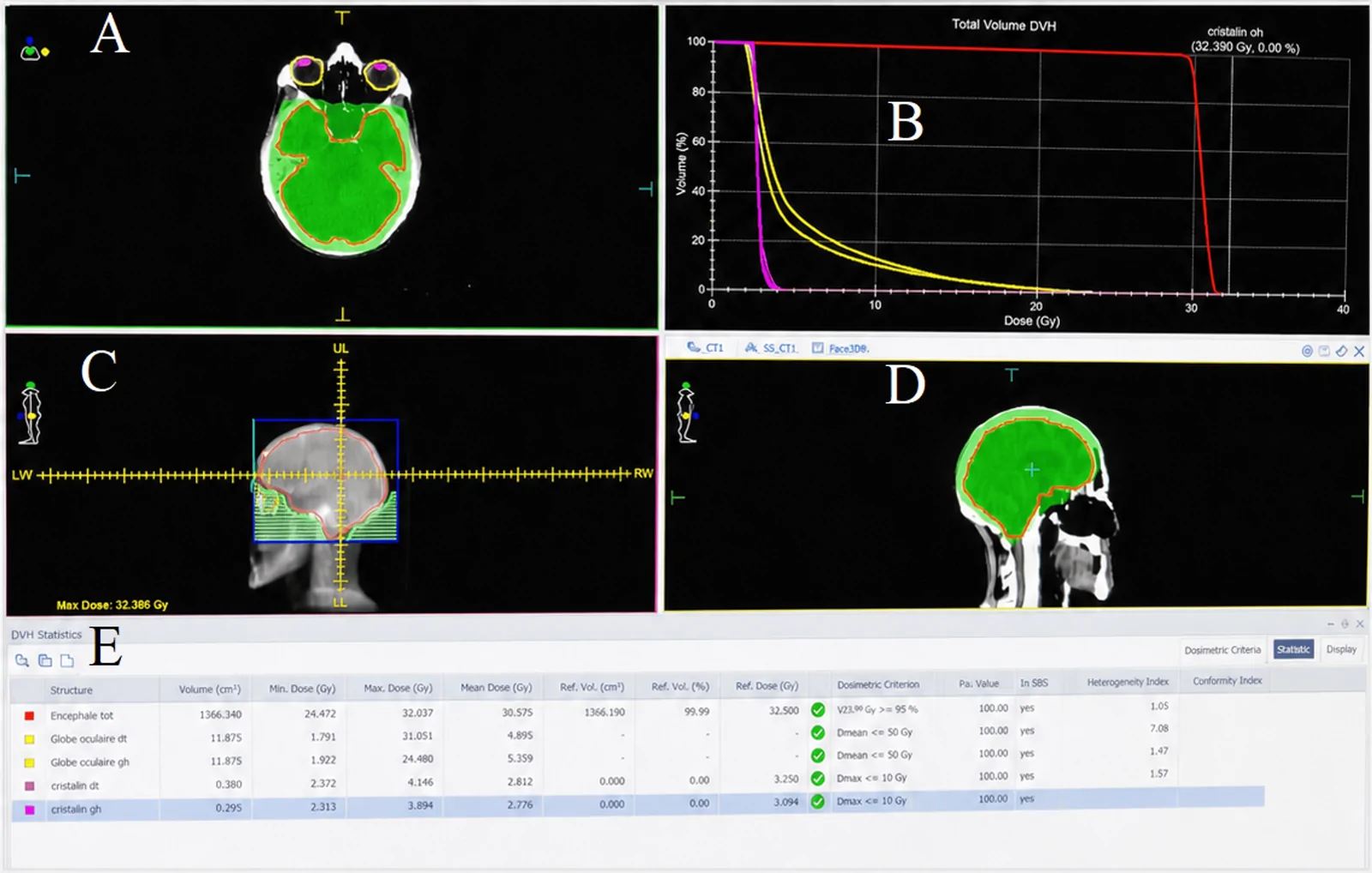
Radiotherapy treatment planning and dose-volume analysis (A) Axial CT section showing delineation of the target brain volume and ocular organs at risk. (B) Dose-volume histogram illustrating dose distribution to the target volume and organs at risk. (C) Coronal reconstruction of the treatment plan showing target volume delineation and isodose distribution. (D) Sagittal reconstruction showing delineation of the target brain volume and dose distribution. (E) Detailed dosimetric analysis displaying the main dose parameters, dosimetric criteria, and heterogeneity and conformity indices.

Management was aimed at combating the complications of the brain metastasis, which could be life-threatening in the short term. This was subsequently combined with systemic chemotherapy (cisplatin plus gemcitabine), with a palliative objective given the disseminated metastatic stage. The patient achieved stable disease and remains alive at six months of follow-up.

## Discussion

The skin is an uncommon site of metastasis in visceral cancers, with an incidence generally reported between 0.6% and 10% in the literature [4]. According to a meta-analysis by Krathen et al., the overall prevalence of cutaneous metastases is approximately 5.3% in patients with cancer [5]. The most frequently implicated primary tumors are those of the lungs, brain, bones, liver, and adrenal glands [6].

In clinical practice, 1% to 12% of patients with lung cancer develop skin metastases, which can manifest in a wide variety of ways (single or multiple nodules, plaques, ulcerated lesions). It is therefore essential that clinicians maintain a high level of suspicion. In some patients, these skin lesions may represent the first sign of previously undiagnosed lung cancer, indicating that the disease is at an advanced stage and that the prognosis is poor [7-9].

Some metastases extend deep into the dermis, reaching the subcutaneous tissues, while others remain superficial. Metastatic tumors can be located anywhere on the skin, but they primarily affect areas near the primary tumor. Metastases can thus be located on the chest (as in our case), back, abdomen, or umbilicus (Sister Mary-Joseph nodule) [10]. 

From an anatomopathological perspective, lung adenocarcinoma currently represents the predominant histological subtype of bronchopulmonary cancers, accounting for approximately 40% to 50% of cases. Its increasing incidence, particularly in both non-smokers and smokers, is attributed to changes in tobacco exposure patterns and evolving characteristics of inhaled carcinogens. Anatomically, it preferentially develops in the peripheral regions of the lung parenchyma. Other histological entities, notably squamous cell carcinoma and small cell lung carcinoma, are less frequent. This distribution is consistent with the updated WHO classification of thoracic tumors [11].

Regarding immunohistochemistry, the identification of the primary origin of cutaneous metastases relies on the use of a panel of epithelial markers and organ orientation. In our observation, the tumor diffusely expressed CK7, with an absence of CK20 expression. This is a profile classically observed in non-small cell lung carcinomas but also in certain tumors of breast, biliary, or gynecological origin [12]. Conversely, the absence of expression of markers specific to pulmonary adenocarcinoma, notably TTF-1 and Napsin A, poses a diagnostic challenge. Although these two markers are considered the most sensitive for identifying lung adenocarcinomas, their sensitivity remains imperfect, with positivity reported in approximately 70% to 80% and 60% to 70% of cases, respectively [13].

Consequently, a significant proportion of lung carcinomas, particularly poorly differentiated or advanced forms, can be doubly negative, as observed in our case. The negativity of TTF-1 and Napsin A can be explained by several mechanisms: tumor dedifferentiation at the metastatic stage, histological variants such as mucinous adenocarcinomas or squamous cell carcinomas, or heterogeneity of antigen expression within the tumor [13-15]. In this context, the interpretation of immunohistochemistry must remain integrated and correlated with clinical and radiological data. The CK7-positive/CK20-negative profile, associated with the presence of a primary lung lesion and the absence of evidence for another primary lesion, remains highly suggestive of a pulmonary origin, even in the absence of TTF-1 and Napsin A expression [14].

To refine the differential diagnosis, some authors recommend the use of complementary markers such as p40 or p63 (in favor of squamous differentiation), GATA3 (breast), CDX2 (digestive origin), or PAX8 (renal or gynecological origin), allowing the exclusion of other possible sources of cutaneous metastases [12]. Table 1 summarizes the main immunohistochemical markers discussed and their associated tumor types. Thus, our observation illustrates the limitations of isolated immunohistochemistry and underlines the importance of a multidisciplinary approach, combining clinical, radiological, morphological, and immunohistochemical data to accurately establish the origin of a cutaneous metastasis.

**Table 1:** Main immunohistochemical markers and associated tumor types TTF-1, thyroid transcription factor-1.

Marker	Associated tumor type	Sensitivity/specificity (approx.)
TTF-1	Pulmonary adenocarcinoma	Sensitivity ~70%-80%
Napsin A	Pulmonary adenocarcinoma	Sensitivity ~60%-70%
p40/p63	Squamous cell carcinoma (pulmonary or other)	Markers of squamous differentiation
GATA3	Breast, urothelial	Excludes breast origin
CDX2	Digestive origin (mainly colorectal)	Excludes digestive origin
PAX8	Renal or gynecological origin (ovary, thyroid)	Excludes these origins

The pre-treatment assessment aims to identify the primary tumor and determine its locoregional and distant spread. It relies primarily on high-resolution chest imaging for localization and morphological characterization of the suspected lung lesion. This is supplemented by thoraco-abdomino-pelvic CT and cerebral MRI to detect any visceral or intracranial metastases. Positron emission tomography combined with CT can be used to evaluate the overall tumor burden and identify subclinical metastatic foci. Histological analysis of the primary or metastatic specimen is systematically included to confirm the tumor nature. This comprehensive assessment is an essential prerequisite for any clinical decision [16,17].

Regarding local management of the cutaneous lesion itself, therapeutic options described in the literature include surgical excision, local radiotherapy, and topical or systemic therapy, depending on the extent of disease and treatment intent (curative, palliative, or purely symptomatic). In our patient, given the exclusively palliative context and extensively disseminated disease, only local wound care was provided, without dedicated surgical or radiotherapeutic treatment of the skin lesion.

Regarding the therapeutic strategy, a multidisciplinary consultation meeting was necessary, and the decision adopted was a combined approach associating WBRT with palliative chemotherapy.

WBRT allows for homogeneous irradiation of the entire cerebral parenchyma, including subclinical micrometastases. The usual hypofractionated regimens (30 Gy in 10 fractions or 20 Gy in 5 fractions) provide a good compromise between efficacy and tolerability, taking into account the patient's overall condition as assessed by the WHO performance status scale. Biologically, irradiation induces DNA double-strand breaks, activates programmed cell death pathways, and alters tumor repair mechanisms. This results in a reduction of intracranial tumor burden, a decrease in vasogenic edema, and an improvement in neurological symptoms [2,18].

Systemic chemotherapy, administered concurrently or sequentially, had a dual therapeutic objective: to ensure overall control of metastatic disease and to achieve a reduction in thoracic tumor volume through a local cytoreductive effect [3].

The choice of cytotoxic agents is based on tumor histology and molecular profile, as well as their potential ability to cross the blood-brain barrier. Their mechanisms of action involve inhibiting DNA replication, disrupting mitosis, and inducing apoptosis through genotoxic lesions and oxidative stress. In this palliative approach, the strategy aims to slow tumor progression, reduce the thoracic mass responsible for local symptoms, and improve clinical status and quality of life [7,13,14].

The combination of radiotherapy and chemotherapy also presents a potential synergistic effect, as radiotherapy can increase tumor permeability and some cytotoxic drugs act as radiosensitizers [3].

However, this strategy requires a rigorous benefit-risk assessment, as WBRT is associated with late neurocognitive side effects, asthenia, and diffuse alopecia, while chemotherapy exposes patients to systemic toxicities (myelosuppression, mucositis, digestive disorders), necessitating appropriate supportive care [3].

Regarding surgery in this case of disseminated lung tumor, it has not demonstrated any effect on overall survival. Metastases indicate systemic dissemination, and surgical resection does not alter the course of the disease, except in exceptional oligometastatic cases where it can be integrated into a multimodal, pseudo-curative strategy [19]. Thus, for multiple brain metastases, current recommendations favor stereotactic radiotherapy or total brain radiotherapy rather than surgery [20]. The same applies to bone metastases, which only benefit from surgery in cases of pathological fractures or spinal cord compression, without impact on survival [21]. On the other hand, extensive lymph node or cutaneous metastases are markers of disseminated disease; surgery is useless for curative purposes and remains reserved for diagnostic or palliative procedures [8,22].

Ultimately, the multimodal palliative strategy aims for optimal symptom control, temporary stabilization of the disease, and maintenance of quality of life.

## Conclusions

Cutaneous metastases of pulmonary origin are a rare but suggestive manifestation of disseminated disease. This case illustrates the diagnostic challenge posed by negative TTF-1 and Napsin A staining, and underscores that their negativity does not rule out a pulmonary origin, particularly in poorly differentiated tumors. Diagnosis in such cases relies on rigorous integration of clinical, radiological, and pathological data.

From a therapeutic standpoint, management in extensively disseminated disease remains essentially palliative. In our patient, a multimodal approach combining WBRT and systemic chemotherapy (cisplatin-gemcitabine) was adopted, with stable disease observed at six months of follow-up. Longer follow-up and a larger case series would be needed to draw conclusions on the impact of this approach on disease control or quality of life.
